# Qualitative Magnetic Resonance Imaging Assessment of the Semimembranosus Tendon in Patients with Medial Meniscal Tears

**DOI:** 10.3390/diagnostics14171962

**Published:** 2024-09-05

**Authors:** Haron Obaid, Adarsh Patel, Emily McWalter, Mark Ernst, Prosanta Mondal, Michael L. Shepel

**Affiliations:** 1 Department of Medical Imaging, Faculty of Medicine, University of Saskatchewan, Saskatoon, SK S7N 5A9, Canada; 2 Department of mechanical Engineering, University of Saskatchewan, Saskatoon, SK S7N 5A9, Canada; 3 Division of Orthopedic Surgery, Department of Surgery, Faculty of Medicine, University of Saskatchewan, Saskatoon, SK S7N 5A9, Canada; 4 Clinical Statistic Unit, Faculty of Medicine, University of Saskatchewan, Saskatoon, SK S7N 5A9, Canada

**Keywords:** semimembranosus, MRI, medial meniscus

## Abstract

Background: To determine if there is an association between semimembranosus tendinosis and medial meniscal tears using MRI. Methods: A retrospective review of knee 3T MRI scans was performed to determine the presence or absence of medial meniscal tears in patients with semimembranosus tendinosis. All studies were interpreted by two musculoskeletal radiologists. Univariate association for the presence of semimembranosus tendinosis and medial meniscal tears was performed with a Chi-square test followed by logistic regression modelling among statistically significant associations. Results: A total of 150 knee MRI scans were reviewed (age 32.8 ± 7.1 years; 70 females). Semimembranosus tendinosis was present in 66 knees (44%) in the patient population. Semimembranosus tendinosis was present in 81% of patients with meniscal tears versus 36% of patients without meniscal tears (*p* < 0.0001). This association remained statistically significant when adjusted for age and sex with an adjusted odds ratio of 7.0 (*p* < 0.0003). Models adjusted for the above covariates and containing the interaction term produced an adjusted odds ratio of 13.0 (*p* < 0.0001) in men, while in women this association was non-significant with an adjusted odds ratio of 2.0 (*p* = 0.42). Conclusions: Subjects with semimembranosus tendinosis were seven times more likely to have medial meniscal tears even when adjusting for sex and age. This could help guide the appropriate postmeniscal repair rehabilitation protocol.

## 1. Introduction

The meniscis are wedge-shaped fibrocartilagenous structures that are situated between the femoral condyles’ and tibial plateau’s weight-bearing structures [1]. Biomechanical studies have shown that the menisci play a major role in the distribution of axial loads over wider articular surface areas by increasing tibiofemoral joint space, stabilizing the knee joint, and lubricating the underlying articular cartilage [2]. Further studies have found that up to 80% of knee loads are transmitted through the menisci [3,4]. As such, the menisci are prone to excessive biomechanical alterations due to axial loading forces and radial hoop stresses [5,6]. Meniscal tears are very common knee pathologies that may result in knee joint instability and degenerative joint disease in the long term [7,8]. The posterior horn of the medial meniscus has a complex capsulotendinous attachment, which includes semimembranosus tendon insertion at the posterior medial meniscocapsular attachment [9]. The semimembranosus muscle has a primary role as a flexor of the knee joint in addition to hip extension [10]. However, the distal tendinous insertion of this tendon onto the tibia and meniscocapsular tissues is complex, with five discrete tendinous arms described [11,12]. Diagnostic MRI imaging is crucial in the clinical settings of meniscal with high diagnostic accuracy compared to arthroscopy [13,14,15,16,17]. MRI has also been shown to be a useful imaging test in the evaluation of tendon pathology [18,19]. 

There is a growing interest in exploring the interplay between the menisci and the semimembranosus muscle to gain a deeper understanding of the implications of biomechanical alterations and overall joint health that may occur as a result of semimembranosus tendon pathology and their effects on the medial meniscus [20,21,22]. Given this unique anatomic and thus resulting biomechanical relationship between the medial meniscus and semimembranosus, we hypothesized that semimembranosus tendinosis could be associated with medial meniscal tears using qualitative MRI imaging.

## 2. Materials and Methods

### 2.1. Ethics

Ethics approval was obtained from our Institutional Ethics Board and is in compliance with local regulations.

### 2.2. Inclusion and Exclusion Criteria

All patients underwent an MRI examination of the knee and met the following criteria: (a) age between 20 and 50 years of age, (b) no previous history of knee trauma, surgery, infection, or metabolic bone disease, and (c) no findings of internal derangement, such as cruciate or collateral ligament tears, on the MRI examination. Exclusion criteria were: (a) all patients who did not meet the above criteria, (b) suboptimal or nondiagnostic MRI images due to motion or metal artefacts, and (c) incomplete MRI imaging due to termination of scan if patients experienced claustrophobia, pain, or discomfort during the MRI examination. The upper limit for the age range was set at 50 years so that age-related degenerative meniscal changes could be avoided [23]. Indications for the MRI scans included symptoms such as pain, limited range of motion, knee swelling and locking. Subjects were identified using our electronic imaging database, Montage (Nuance^®^ mPower, Burlington, MA, USA). Patients’ identifying information was kept anonymous through use of a master list and data anonymization tool.

### 2.3. MRI Imaging Technique

All subjects were scanned on a 3TMRI scanner (Magnetom^®^ Skyra, Siemens Healthcare, Erlangen, Germany) using Software Numaris/4, Version Syngo MR E11, and an 18-channel receiver-only body array coil. The knee MRI protocol consisted of a high-resolution coronal PD Sampling Perfection with Application-optimized Contrasts using different flip angle Evolution (SPACE) (TR/TE = 1200/27, slice = 0.75 mm, field of view = 160 mm), a coronal proton density (PD) with fat saturation (FS) (TR/TE = 2500/33, slice = 3 mm, field of view = 150 mm), an axial PD FS (TR/TE = 3500/33, slice = 3 mm, field of view = 160 mm), a sagittal PD FS (TR/TE = 4310/33, slice = 3 mm, field of view = 160 mm), and a sagittal T1 (TR/TE = 971/12, slice = 3 mm, field of view = 160 mm).

### 2.4. Qualitative Image Analysis

**Figure 1:**
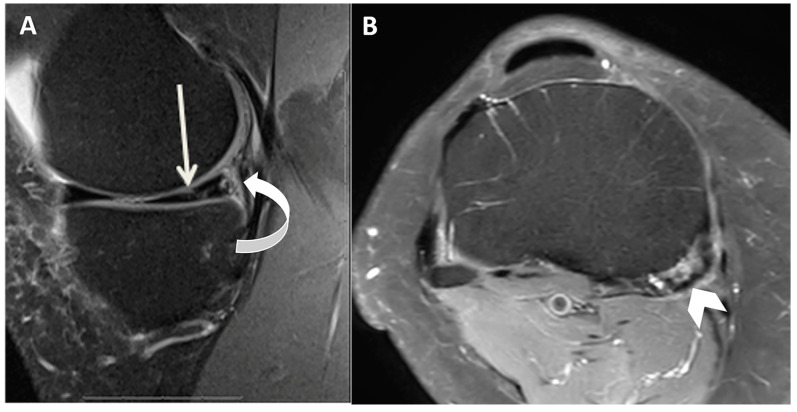
MRI of the right knee of a 31-year-old male patient who presented with medial-sided knee joint pain. Sagittal Proton Density Fat Saturated (**A**) and axial Proton Density Fat Saturated (**B**) MRI images demonstrate medial meniscal tear (arrow), posterior mensicocapsular separation (curved arrow), and semimembranosus tendinosis (chevron).

**Figure 2:**
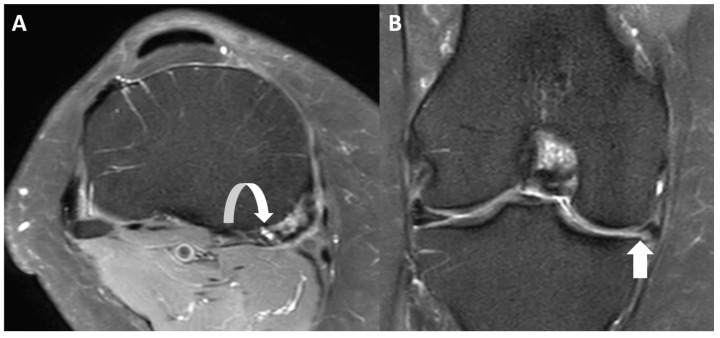
MRI scan of the right knee of a 40-year-old male patient who presented with medial-sided knee pain and knee locking. (**A**) Axial Proton Density Fat Saturated MRI image demonstating a focal fluid-filled defect at the insertion of the semimembranosus tendon in keeping with a focal tear (curved white arrow). (**B**) Coronal Proton Density Fat Saturated MRI image demonstrating undersurface tear of the body of the medial meniscus (white arrow).

**Figure 3:**
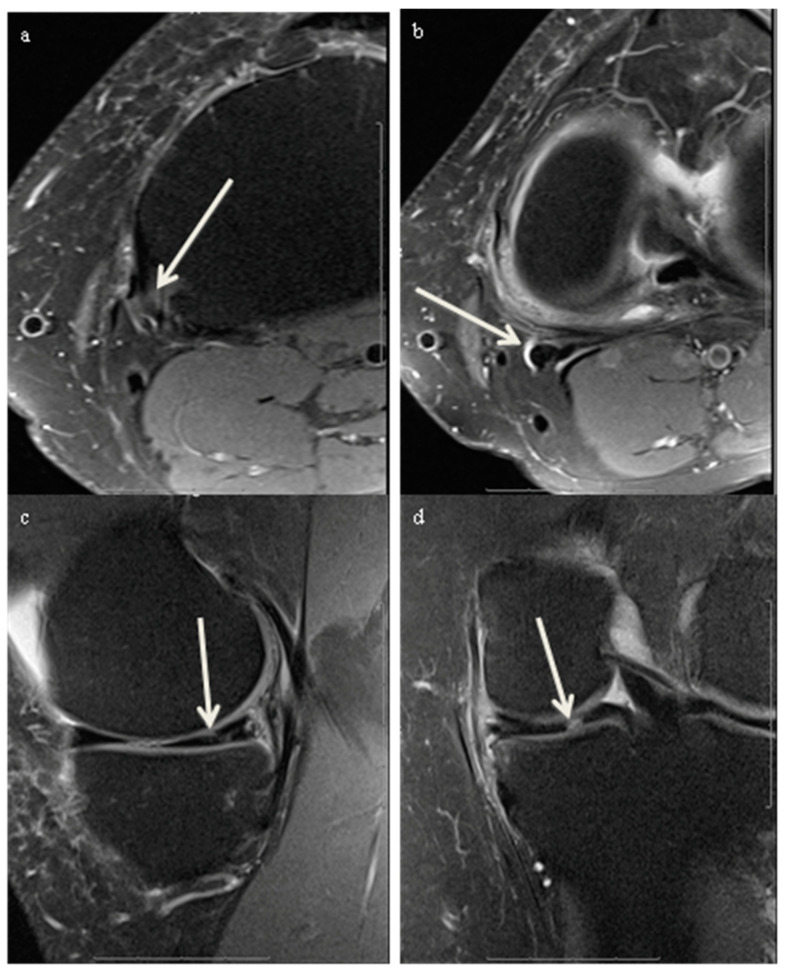
MRI scan of the left knee of a 34-year-old male patient who presented with medial sided knee pain and swelling. (**a**) Axial Proton Density Fat-Saturated MRI image, which demonstrated semimembranosus tendinosis (white arrow). (**b**) Axial Proton Density Fat Saturated image superior to (**a**) demonstrating presence of fluid within the semimembranosus bursa in keeping with bursitis (white arrow). (**c**,**d**) Sagittal and coronal Proton Density Fat Saturated MRI images, respectively, which demonstrate complex medial meniscal tear with horizontal and radial components involving the body and posterior horn (white arrow).

All images were reviewed on a Picture Archiving and Communication System (Philips IntelliSpace PACS 4.4.541.5) and displayed on high-resolution radiology diagnostic monitors (Coronis Fusion 6-megapixel LED Barco monitors MDCC-6230, Barco NV, Kortrijk, Belgium). Two fellowship-trained musculoskeletal (MSK) radiologists, with 22 and 19 years of experience, assessed the medial menisci for the presence of meniscal tears and the semimembranosus tendon for tendinosis and associated abnormalities such as tears and bursitis. The medial meniscus was assessed for the presence or absence of meniscal tears using a binary 0–1 system, where 0 refers to the absence of meniscal tears and 1 refers to the presence of meniscal tears (Figure 1). A meniscal tear was defined as a fluid-filled cleft breaching one or two articular surfaces in the meniscus seen on two slices and two planes [17]. The presence or absence of semimembranosus tendinosis was assessed using a binary 0–1 system, where 0 refers to normal tendon and 1 refers to tendinosis. Tendinosis was defined by the presence of tendon heterogeneity, increased signal within the tendon, striation of the tendon, peritendinous edema, or tearing (Figure 1) [24]. Semimembranosus tears were defined as focal fluid-filled defects in the semimembranosus tendon (Figure 2) [24]. Semimembranosus bursitis was described as a fluid-filled sac in the expected anatomic location of the semimembranosus bursa around the distal semimembranosus tendon (Figure 3). Interreader agreement was achieved in all cases for meniscal tears and tendinosis by consensus between the two readers.

### 2.5. Statistical Analysis

The medial meniscus was assessed for tears versus no tears, and the presence of semimembranosus tendinosis was tested in univariate association by Chi-square testing. This was followed by logistic regression modelling to assess age and sex as potential confounders of the primary relationships. Among statistically significant associations, interactions between covariates and the key predictor of interest were examined. Analysis was carried out using packaged statistical software (IBM SPSS Statistics for Windows, Version 20.0. Armonk, NY, USA: IBM Corp).

## 3. Results

A total of 1000 knee MRI scans were initially reviewed, and 150 knee MRI scans met the inclusion criteria for the study. The average age of the patients was 32.8 ± 7.1 years. There were 70 females and 80 males. Laterality was observed in 77 right knees (51%), and 73 left knees (49%). 

**Figure 4:**
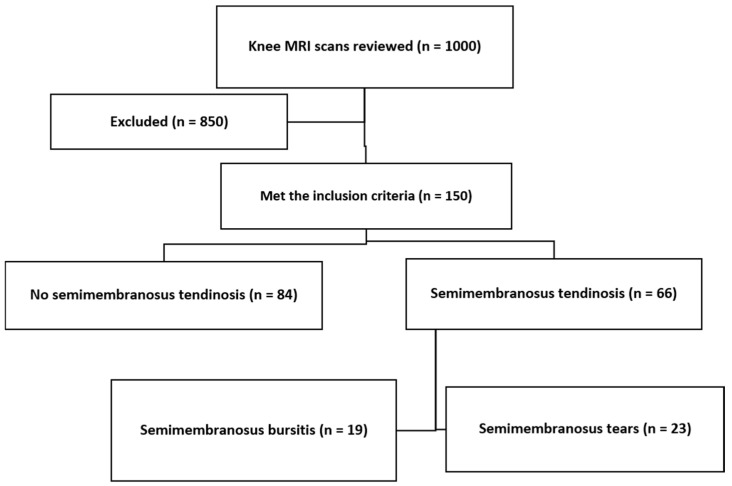
Flowchart of patients demonstrating semimembranosus changes, including tendinosis, tears, and bursitis.

Semimembranosus tendinosis was present in 66 knees (44%). In 35% of these patients, there were associated semimembranosus tears, and in 29% there was associated semimembranosus bursitis (Figure 4).

Subjects with medial meniscal tears were statistically significantly more likely to have semimembranosus tendinosis than those without tears (81% vs. 36%, *p* < 0.0001), as shown in Table 1. 

Meniscal tears were seen more frequently (57.8%) in the posterior horn of the medial meniscus (Table 2). The distribution of the meniscal tear patterns was longitudinal (*n* = 10), horizontal (*n* = 7), complex (*n* = 6), and radial (*n* = 3).

The results of univariate and logistic regression analysis for medial meniscal outcomes showed that individuals with medial meniscal tears were seven times more likely to have semimembranosus tendinosis when adjusted for age and sex (*p* < 0.0003) (Table 3). There was a statistically significant interaction between semimembranosus tendinosis and sex, suggesting that the relationship between semimembranosus tendinosis and meniscal tears is not consistent in men and women. Models adjusted for the above covariates and containing the interaction term produced an adjusted odds ratio of 13.0 (*p* < 0.0001) in men, while in women, this association was nonsignificant, with an adjusted odds ratio of 2.0 (*p* = 0.42).

## 4. Discussion

The menisci play a major role in the knee joint biomechanics, aiding its stability and allowing even distribution of axial forces across the articular surface [25,26]. Studies have shown a complex anatomical and functional relationship between the medial meniscus and the distal semimembranosus tendon insertion [9,21]. The function of the semimembranosus is protective of the medial meniscus due to its complex anatomy with five insertion bands, namely, anterior, direct, capsular, inferior, and the oblique popliteal ligament [27]. These tendinous bands intertwine with the posterior oblique ligaments, adding stability for the posteromedial aspect of the knee and the medial meniscus [28]. The semimembranosus is responsible for protecting the posterior horn of the medial meniscus by retracting the medial meniscus during knee flexion, preventing it from entrapment in the tibiofemoral joint line [28]. It could therefore be conceivably argued that abnormal semimembranosus may result in failure of meniscal retraction and therefore entrapment in the joint line and meniscal tears. Semimembranosus tendinosis occurs due to friction and repetitive eccentric tendon loading, which can lead to degenerative changes in the tendon and its insertions [12]. Meniscal tears could occur due to trauma, meniscal entrapment, meniscal impingement, or meniscal degeneration [23,29].

In this study, semimembranosus tendinosis was identified in 44% of our patient population, which is consistent with the previously published rates of 44.8–52.6% [10,30]. In addition, our study demonstrated a strong association between the presence of semimembranosus tendinosis and medial meniscal tears, whereby individuals with semimembranosus tendinosis were seven times more likely to have medial meniscal tears even when adjusted for age and sex (*p* < 0.0003). Semitendinosis tears and bursitis were seen in 35% and 29%, respectively. Mensical tears were seen most frequently in the posterior horns (57.8%). Previous studies have found that the prevalence of tears affecting the posterior horn of the medial meniscus is 56% [31]. This was thought to be related to the anatomic characteristics of the posterior horn being wider and larger in size, which results in more axial distribution of weight loading and shock absorbing across the posterior horn of the medial meniscus, making it more prone to meniscal tears [29].

The association between semimembranosus tendinosis and medial meniscal tears may be explained by the complex meniscal biomechanics and local anatomy [22,26,30]. The semimembranosus insertion onto the posterior horn of the medial meniscocapsular attachment is predominantly reinforced by the capsular arm of the tendon, which provides the additional function of helping to retract the posterior horn of the medial meniscus posteriorly during active knee flexion to avoid meniscal entrapment in the joint line [20,22]. Within this paradigm, the association between semimembranosus tendinosis and medial meniscal tears may have a basis in the underlying anatomy and resulting biomechanics of meniscal motion. Kinematic MRI studies of meniscal motion by Thompson et al. and Vedi et al. have shown that the medial meniscus undergoes posterior translation during knee flexion [25,26]. 

To the best of our knowledge, the sex-dependent association between the presence of semimembranosus tendinosis and meniscal changes has never been previously demonstrated. However, numerous studies have previously demonstrated sex-related differences in lower extremity biomechanics. Malinzak et al. showed less peak knee flexion and flexion excursion in female runners, while Ferber et al. demonstrated differences in nonsagittal plane motion in female runners with a greater peak knee abduction angle and a more abducted knee in all positions of gait and a trend towards greater external rotation [32,33]. Studies have shown that the connective tissues of men and women are physiologically different, with muscle elasticity being stiffer in men than women, resulting in greater muscle compliance in women than men [34]. In addition, anatomical factors such as greater peak knee extension in men than women can lead to different mechanical profiles and hamstring flexibility between men and women [35]. Sex-related gait analysis conducted by Cho et al. demonstrated less ground impact in the initial contact phase of gait with resultant decreased activation of the knee flexors and increased effort during knee extension in the push-off phase in women [36]. It is suggested that sex-related biomechanical differences such as these, especially differences in nonsagittal plane motion, may play a role in the higher incidence of patellofemoral and iliotibial band syndrome in women [37]. Conversely, the same sex-related biomechanical differences may predispose males to developing semimembranosus tendinosis. In addition, this observed difference parallels the higher rate of meniscal tears seen in males, further suggesting that semimembranosus dysfunction may play a role in the development of meniscal degeneration and tears [8]. 

Studies have shown that early surgical management of meniscal tears is beneficial to the patients in terms of symptomatic relief as well as long-term therapeutic advantages to prevent the development of osteoarthritis and cartilage degeneration [38]. Studies have also found that the status of the semimembranosus tendon is crucial in facilitating knee joint rehabilitation after meniscal repair surgery [39]. The association between semimembranosus tendinosis and medial meniscal tears could result in recurrent meniscal tears after meniscal repair. Therefore, treatment strategies with a hamstring muscle rehabilitation protocol aimed at improving semimembranosus could help ameliorate postoperative outcomes and reduce the risk of recurrent meniscal tears for this patient population [39].

Our study has several limitations. The retrospective nature of the study may have led to an intentional selection bias; however, with our strict inclusion criteria, we believe that this bias has been minimized. The diagnosis of meniscal tears and semimembranosus tendinosis was based on imaging tests rather than surgery; however, MRI has been proven to show high accuracy rates for meniscal and tendon pathologies. In addition, if the selection criteria were to be limited to those patients with arthroscopic surgery, this would have significantly reduced the study sample size. This retrospective study design identified an association between semimembranosus tendinosis and medial meniscal tears. To establish a causal relationship between semimembranosus tendinosis and medial meniscal tears, an appropriately designed research model would be recommended. Finally, although this is a single-centre study, we believe that the double-read consensus-based system by two fellowship-trained musculoskeletal radiologists has strengthened the validity of the results.

## 5. Conclusions

Our study examined the relationship between semimembranosus and meniscal tears using qualitative MRI and showed that individuals with semimembranosus tendinosis were seven times more likely to have medial meniscal tears, which may be explained by the complex anatomic relationship of this tendon and the medial meniscus. The results of this study could help shed light on the interplay between semimembranosus and medial meniscal tears to help guide appropriate semimembranosus muscle therapeutic rehabilitation programmes in postoperative meniscal surgery. This is an area of growing interest, and future studies utilizing emerging imaging techniques such as kinematic MRI imaging, Diffusion Tensor Imaging (DTI), and MRI and ultrasound elastography could be performed to help explore the gender variation between men and women as well as understand the pathophysiological causes of semimembranosus tendinosis and its effect on the complex anatomy of the posteromedial corner of the knee.

## Figures and Tables

**Table 1:** The status of the medial meniscus and semimembranosus tendon were tested in univariate association using Chi-square.

Medial Meniscus—Tear vs. No Tear			
Semimembranosus Tendinosis, N (%)	Tear N = 26	No Tear N = 124	*p* Value *
Yes, N = 66 (44)	21 (81)	45 (36)	<0.0001
No, N = 84 (56) Total = 150	5 (19)	79 (63)	

Numbers in brackets represent percentiles. * Implies statistical significance.

**Table 2:** Distribution of meniscal tears in the medial meniscus.

Anatomic Area of the Meniscus	Number of Tears
Posterior horn	15 (57.8)
Body	8 (30.7)
Anterior horn	3 (11.5)

Numbers in brackets represent percentiles.

**Table 3:** Logistic regression analysis for semimembranosus tendinosis in patients with meniscal tears. The results were adjusted for age and sex. 95% confidence intervals in brackets.

Outcome: Medial Meniscus Tear				
	Crude OR	*p* Value	Adjusted OR *	*p* Value
Semimembranosus Tendinosis	6.0 (2.4, 14.9)	<0.0001	7.0 (2.4, 20.2)	<0.0003

* Adjusted for age and sex.

## Data Availability

Data are available upon reasonable request.
